# Body height and spinal pain in adolescence: a cohort study from the Danish National Birth Cohort

**DOI:** 10.1186/s12891-023-07077-3

**Published:** 2023-12-11

**Authors:** Anne Cathrine Falch-Joergensen, Per Kragh Andersen, Esben Budtz-Jorgensen, Lise Hestbaek, Katrine Strandberg-Larsen, Anne-Marie Nybo Andersen

**Affiliations:** 1 Section of Epidemiology, Department of Public Health, Faculty of Health and Medical Science, University of Copenhagen, Oster Farimagsgade 5, Box 2099, Copenhagen K, DK-1014 Denmark; 2 Section of Biostatistics, Department of Public Health, Faculty of Health and Medical Science, University of Copenhagen, Copenhagen K, Denmark; 3 Department of Sport Science and Clinical Biomechanics, University of Southern Denmark, Odense, Denmark

**Keywords:** Spinal pain, Back pain, Epidemiology, Adolescence, Body height, Growth

## Abstract

**Objectives:**

To investigate how body height and trajectories of height from infancy through childhood and adolescence were associated with spinal pain in pre- and late adolescence.

**Methods:**

This prospective study included 43,765 individuals born into The Danish National Birth Cohort (DNBC) from 1996 to 2003. DNBC-data were linked with health and social data identified from Statistics Denmark registers. Spinal pain was self-reported in both the 11-year- and 18-year follow-up of DNBC and classified according to severity. Body height was measured from birth and onwards and further modelled as distinct developmental height trajectories by using latent growth curve modelling. Associations were estimated by using multinomial logistic regression models.

**Results:**

Taller body height in childhood and adolescence was associated with approximately 20% increased likelihood of spinal pain in pre- and late adolescence among girls compared to their peers in the normal height group. For boys, taller body height was associated with spinal pain by late adolescence only. Spinal pain in pre-adolescence almost doubled the likelihood of spinal pain in late adolescence regardless of body height at age 18. Height trajectories confirmed the relationship for girls with the tall individuals being most likely to have spinal pain in both pre- and late adolescence.

**Conclusion:**

Tall body height during childhood and adolescence predisposes to spinal pain among girls in both pre-and late adolescence, and among boys in late adolescence. Body height is a contributing factor to the pathogenesis of spinal pain in adolescence; however, the mechanisms may be related to growth velocity, but for now uncertain.

**Supplementary Information:**

The online version contains supplementary material available at 10.1186/s12891-023-07077-3.

## Introduction

Spinal disorders constitute an enormous global burden with adverse societal and personal consequences [1], and low back and neck pain has been documented as leading causes of disabilities globally [2]. Evidence suggests spinal pain to have its onset already in childhood and the prevalence to reach adult levels around age 18 [3–7]. In addition, pediatric onset of spinal pain has been suggested to predict spinal pain in later life, and thus, spinal pain can be a long-term experience [3]. Etiology of spinal pain may be found in a complex interplay between psychosocial, environmental, and biological components. Psychosocial and environmental factors such as depressive symptoms [8, 9], stress and poor general well-being [10], as well as living in socioeconomically disadvantaged families [11] have been suggested as potential risk factors for the development of spinal pain in young people. However, there is little evidence to suggest that biological and genetical factors in terms of body height and growth during childhood and adolescence are associated with back pain in adolescents [12, 13].

Body height is hardly modifiable and mainly influenced by environmental factors and genetic predispositions [14–17]. Despite limited possibilities for intervention, body height and growth patterns are important aspects in the understanding of the interplay and impact of risk factors predisposing to spinal pain already in adolescence and identification of particularly vulnerable subgroups may guide personalized prevention.

Therefore, the overall aim of this study was to investigate how body height from birth up to adolescence were associated with spinal pain in pre-adolescence and late adolescence. Specifically, we aimed to first investigate body height at different ages from birth through childhood to adolescence in relation to spinal pain in pre-adolescence (11–12 years of age) and subsequently to spinal pain in late adolescence (18 years of age). Furthermore, we investigated developmental patterns of height and related these distinct height trajectories to spinal pain in 11-12-year-olds and 18-year-olds, respectively.

## Methods

### Study population

**Fig. 1:**
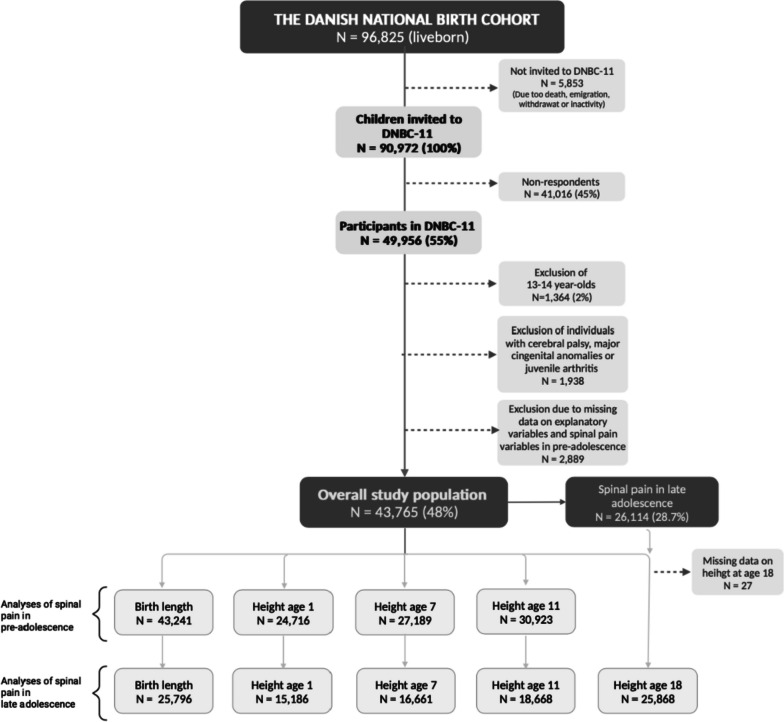
Flow chart of the study population and additional analysis samples

This longitudinal study applied data from The Danish National Birth Cohort (DNBC), nested within the nationwide population of Denmark. In brief, DNBC is a population-based cohort including 98,825 children born between 1996 and 2003, followed from birth and through childhood and young adulthood. DNBC is described in detail elsewhere (www.dnbc.dk) [18]. The overall study population consisted of 43,765 individuals 11–12 years of age that participated in the 11-year follow-up of DNBC (DNBC-11) and who had at least one height measure reported from birth to age 11. Selection mechanisms are depicted in the flow chart in Fig. 1. Since the study included height exposures at different ages as well as spinal pain outcomes in both pre-adolescence and late adolescence, we defined specific analysis samples according to the respective analyses to obtain the highest power for each analysis (see Fig. 1).

DNBC-data was linked to Danish national registers through the unique personal identification number assigned to all persons with a permanent residence in Denmark [19]. All registries applied were available and processed at Statistics Denmark, and all data were pseudonymized. Approval of the study was obtained from the Danish Data Protection Agency through the joint notification of The Faculty of Health and Medical Sciences at The University of Copenhagen (SUND-2017-09) and the DNBC Steering Committee (2017-23).

### Height measures

Length at birth was retrieved from the Danish Medical Birth Registry [20]. The remaining height measures at age 1, 7, 11 and 18 were obtained from DNBC data collections. Height data were carefully cleaned for errors before any further data management and height measures within the range of -5 to 5 standard deviations (SD) were accepted [21]. Height at age 1 was mother-reported in the 18-months follow-up of DNBC and based on the registrations made by the general practitioner in ‘The Child’s Book’ in connection with routine preventive health visits scheduled at 5 and 12 months. We exclusively allowed inclusion of measurements reported between 11 and 13 months. Height at age 7, 11 and 18 were accepted if reported within the given year, and were further categorized into three percentile groups, calculated by sex. The group of normal height consisted of the 20th to 80th percentile, low height was defined as < 20th percentile, and the > 80th percentile was defined as tall height.

For latent growth curve modeling (LGCM), we modelled height z-scores to enable comparisons across age and sex. Z-scores were calculated using the *Zanthro package* in STATA with standardization according to the WHO-reference and included age in months [22].

### Spinal pain

DNBC-11 included self-reported information on frequency and intensity of neck pain, mid back pain and low back pain. The questions were adopted from The Young Spine Questionnaire (YSQ) designed and validated to measure neck, mid back and low back pain in 9–11 year-olds [23]. Children were asked to report their pain frequency and intensity ranging from 0 to 6 based on the Faces Pain Scale-Revised (FPS-r)) [23, 24]. For each spinal region we combined pain frequency and intensity and trichotomized into *no pain, moderate pain, or severe pain* (Supplementary File 1). For all spinal regions, severe pain was defined as pain of 4 or more on FPS-r and occurring at least ‘once in a while’, and no pain was defined as pain up to 2 on FPS-r occurring ‘once in a while’ or ‘once or twice’. Moderate pain covered the remaining combinations. Subsequently, the main outcome of interest *Spinal pain* was constructed as a composite measure including all spinal regions categorized according to severity. The applied definition is directly adapted from our previous work and documented in detail elsewhere [11]. Information on spinal pain at age 18 was obtained from DNBC-18, which included a similar frequency scale as DNBC-11, however, the intensity scale ranged from 0 to 10 according to the Numeric Pain Rating Scale (NRS). Since no valid method exists to transfer NRS to FPS-r for adolescents, we based the definition of spinal pain in DNBC-18 on a study on elderly people in nursing homes [25], supported by several sensitivity analyses testing the different combinations of intensity and frequency of spinal pain. The applied measures of spinal pain in pre- and late adolescence are illustrated in Supplementary File 1.

### Covariates

Potential confounders were selected a priori, and identified using the methods of causal diagrams (Depicted in Supplementary File 2) [26]. Information on sex was derived from DNBC-11. Parity (nulliparous/parous) and gestational age (term/preterm) were obtained from The Danish Medical Birth Registry [20]. Educational level was obtained from The Danish Population’s Education Register [19], and operationalized as the highest ongoing or completed education of the parents attained at childbirth and was categorized into three groups according to the International Standard Classification of Education (ISCED) 2011: low (ISCED 0–2), medium (ISCED 3–4) and high (ISCED 5–8) [27]. Equivalized household income at the year before childbirth was based on disposable household income extracted from The Income Statistics Register [28]. We divided disposable household income by an equivalence factor corresponding to the modified OECD scale [29]. Equivalized household income was further categorized into quartiles by year relative to all mothers giving birth in the given year. Finally, we created an indicator of pubertal maturation (i.e., having reached pubertal growth spurt) (yes/no) at time of completion of DNBC-11. A Tanner stage of 2 or more for girls and Genital stage of 4 or more for boys indicated that the pubertal growth spurt was reached [30].

### Statistical analyses

Statistical analyses were performed in STATA V. 16.1. To estimate all associations between body height and spinal pain, we used multinomial logistic regression models to calculate crude and adjusted relative risk ratios (RRR) and corresponding 95% confidence intervals (CI) (see Supplementary File 3 for interpretation). Children with no spinal pain were considered the reference outcome in all analyses, and dependency between siblings in the sample was taken into account by applying a robust standard error estimator [31]. We tested and identified sex-differences between sex and height at the different ages by evaluating first-order interactions using Wald-tests, hence, included the interaction between sex and height in each model and further adjusted for the main effects of the identified potential confounders.

In sensitivity analyses, we applied height as a continuous variable in 5 cm intervals after first testing for linearity. Secondly, we performed analyses using neck, mid, and low back pain as separate outcomes. Thirdly, in a sub-analysis of height at age 11 and spinal pain in pre-adolescence, we accounted for pubertal maturation. Lastly, to evaluate the extent to which the results may have been affected by selection forces, we conducted a loss-to-follow-up analysis and subsequently applied inverse probability weighting (IPW) to account for potential selection bias as to handle potential bias arising from exclusion of missing data [32, 33]. Predictor variables in IPW corresponded those included in the loss-to-follow-up analysis.

#### Identification and analysis of height trajectories

LGCM was used to estimate developmental height patterns from childbirth through childhood to adolescence and their relation to spinal pain in adolescence. The analysis was conducted as a two-step process, first by estimating height trajectories with height included as z-scores as a function of age [34]. A priori, we had decided to look at the different height trajectories separately for boys and girls, however, we observed a striking similarity between the two trajectory models, and decided, also supported by a likelihood ratio test, that the modelling of the trajectories should not be separated by sex. The height trajectories were subsequently included as an interaction term between height and sex as described above. Secondly, multinomial logistic regression models were used to calculate associations with spinal pain in pre- and late adolescence [34]. The association was further analyzed separately for each spinal region in a sensitivity analysis. Supplementary File 4 includes detailed description of LGCM.

## Results

The tallest individuals differed from their shorter peers by being more likely to be born to term and from families of higher socioeconomic status (Table 1). On average, boys were of taller height from birth to age 7 (Supplementary File 5). At 11 years, mean height of girls and boys approached equal levels. However, from age 11 to age 18 boys grew more and reached significant taller height than girls. Prevalence of severe spinal pain in pre-adolescence was 12% and 21% in late adolescence with the highest prevelance among girls (Supplementary File 6).

**Table 1:** Characteristics of individuals included in the sample with information on height at age 11, according to height group at age 11 (The Danish National Birth Cohort, 1996–2003, *N* = 30,923)

Characteristics	N (%)	Low height group (< 20%)	Normal height group (20–80%)	Tall height group (> 80%)
Total	30,923 (100)	6,740 (21.8)	18,882 (61.1)	5,301 (17.1)
Child’s sex				
Boys	14,962 (48.4)	3,448 (51.2)	8,906 (47.2)	12,608 (49.2)
Girls	15,961 (51.6)	3,292 (48.8)	9,976 (52.8)	2,693 (50.8)
Gestational age				
Term	29,335 (94.9)	6,284 (93.3)	17,972 (95.2)	5,079 (95.8)
Preterm (< 37 weeks)	1,588(5.1)	456 (6.8)	910 (4.8)	222 (4.2)
Parity				
Nulliparous	15,044 (48.7)	3,282 (48.7)	9,135 (48.4)	2,627 (49.6)
Parous	15,879 (51.4)	3,458 (51.3)	9,747 (51.6)	2,674 (50.4)
Parental educational level				
High	20,057 (64.9)	4,321 (64.1)	12,323 (65.3)	3,413 (64.4)
Medium	10,165 (32.9)	2,230 (33.1)	6,167 (32.7)	1,768 (33.4)
Low	701 (2.3)	189 (2.8)	392 (2.1)	120 (2.3)
Equivalized household income				
4th quartile (highest)	10,482 (33.9)	2,138 (31.7)	6,495 (34.4)	1,849 (34.9)
3rd quartile	9,087 (29.4)	1,988 (29.5)	5,491 (29.1)	1,608 (30.3)
2nd quartile	7,121 (23.0)	1,618 (24.0)	4,366 (23.1)	1,137 (21.5)
1st quartile (lowest)	4,233 (13.4)	996 (14.8)	2,530 (13.4)	707 (13.3)

a Variables were analysed with the chi-squared test of heterogeneity. Chi-squared tests were statistically significant for all variables except for parity

Loss-to-follow-up analyses revealed that the study participants were more often girls, from families of higher socioeconomic status, had non-smoking mothers during pregnancy, and from urban areas compared to those lost to follow-up. This was slightly more significant among those with further follow-up in late adolescence (Supplementary File 7). Nonetheless, applying IPW to account for selection both into the cohort and from attrition had no essential impact on the estimates in any of the analyses included in this study (data not shown).

### Association between body height and adolescence spinal pain

Results from adjusted regression analyses showed that among girls, tall body height during childhood and adolescence was directly associated with moderate and severe spinal pain in pre-adolescence as well as late adolescence (Table 2). The tallest group at age 11 had an increased likelihood of severe spinal pain in pre-adolescence of 26% (RRR: 1.26, 95% CI: 1.11–1.43) relative to no pain and compared to their peers in the normal height group, whereas those in lowest height group were less likely to have spinal pain. The same pattern was observed for height at age 7 and spinal pain in pre-adolescence, and for height at age 11 in relation to spinal pain in late adolescence (Table 2). This dose-response trend was confirmed in sensitivity analyses analyzing height as a continuous variable (Supplementary File 8). Among boys, no definite associations were observed in pre-adolescence. Nevertheless, for boys we observed both tall height at age 11 (RRR: 1.44, 95% CI: 1.19–1.75) and at age 18 (RRR: 1.24, 95% CI: 1.06–1.46) to be associated with spinal pain in late adolescence (Table 2), as did we observe a tendency of a dose-response effect for boys. Additional adjustment for pubertal maturation had no effect on the estimates for boys; however, it weakened the estimates for girls (Supplementary File 9). The prevailing spinal pain regions were the mid and low back for girls, whereas no consistent pattern was observed for boys (data not shown).

**Table 2:** Adjusted relative risk ratio (RRR) of spinal pain in pre-adolescence and late adolescence, respectively, according to body heights at different ages, analyzed as separate models and as the interaction between sex and height (The Danish National Birth Cohort, born 1996–2003)

	Girls^a^			Boys^a^		
	No. of cases Moderate/Severe	Moderate pain RRR (95% CI)	Severe pain RRR (95% CI)	No. of cases Moderate/Severe	Moderate pain RRR (95% CI)	Severe pain RRR (95% CI)
**SPINAL PAIN IN PRE-ADOLESCENCE** ^b^						
** Birth length (*N* = 43,241)**						
Low birth length	1,769/724	0.98 (0.91–1.05)	0.87 (0.79–0.96)	1,668/546	0.99 (0.92–1.06)	0.99 (0.88–1.11)
Normal birth length	4,342/2,023	Ref.	Ref.	3,613/1,190	Ref.	Ref.
Long birth length	821/358	1.01 (0.92–1.11)	0.95 (0.84–1.08)	617/263	0.89 (0.81–0.99)	1.16 (1.00-1.34)
** Body height at age 1 (*N* = 24,716)**						
Low height	912/381	0.98 (0.89–1.07)	0.93 (0.81–1.05)	923/319	1.00 (0.91–1.10)	1.07 (0.93–1.24)
Normal height	2,493/1,100	Ref.	Ref.	1,765/579	Ref.	Ref.
Tall height	594/293	1.07 (0.96–1.20)	1.20 (1.03–1.38)	537/171	1.04 (0.93–1.17)	1.02 (0.85–1.23)
** Body height at age 7 (*N* = 27,189)**						
Low height	839/345	0.94 (0.85–1.03)	0.86 (0.75–0.98)	860/247	1.06 (0.96–1.16)	0.90 (0.77–1.05)
Normal height	2,600/1,167	Ref.	Ref.	2,270/763	Ref.	Ref.
Tall height	724/360	1.15 (1.04–1.28)	1.27 (1.11–1.45)	689/230	1.10 (0.99–1.22)	1.09 (0.93–1.29)
** Body height at age 11 (*N* = 30,923)**						
Low height	929/346	0.88 (0.81–0.97)	0.78 (0.68–0.88)	998/305	1.05 (0.96–1.15)	0.95 (0.83–1.09)
Normal height	2,977/1,258	Ref.	Ref.	2,465/834	Ref.	Ref.
Tall height	875/395	1.19 (1.08–1.30)	1.26 (1.11–1.43)	733/270	1.04 (0.94–1.15)	1.14 (0.98–1.32)
**SPINAL PAIN IN LATE ADOLESCENCE** ^**c**^						
** Birth length (*N* = 25,796)**						
Low birth length	1,365/1,069	0.93 (0.85–1.02)	0.90 (0.82–0.99)	825/276	1.04 (0.94–1.16)	0.84 (0.72–0.98)
Normal birth length	3,488/2,717	Ref.	Ref.	1,665/663	Ref.	Ref.
Long birth length	659/491	1.05 (0.93–1.18)	1.00 (0.88–1.14)	325/137	1.00 (0.86–1.15)	1.06 (0.87–1.29)
** Body height at age 1 (***N*** = 15,186)**						
Low height	735/580	0.94 (0.84–1.06)	0.96 (0.85–1.09)	468/160	0.99 (0.87–1.14)	0.87 (0.71–1.07)
Normal height	2,080/1,601	Ref.	Ref.	868/335	Ref.	Ref.
Tall height	465/414	1.01 (0.87–1.16)	1.18 (1.01–1.36)	280/110	1.11 (0.94–1.31)	1.13 (0.89–1.43)
** Body height at age 7 (*N* = 16,661)**						
Low height	710/545	0.94 (0.84–1.05)	0.95 (0.84–1.08)	390/135	0.88 (0.77–1.01)	0.82 (0.67–1.01)
Normal height	2,194/1,665	Ref.	Ref.	1,154/426	Ref.	Ref.
Tall height	545/467	1.09 (0.95–1.24)	1.23 (1.06–1.41)	338/137	1.11 (0.96–1.28)	1.22 (0.99–1.50)
** Body height at age 11 (*N* = 18,668)**						
Low height	819/585	0.91 (0.82–1.02)	0.82 (0.73–0.93)	465/155	0.94 (0.83–1.07)	0.83 (0.68–1.01)
Normal height	2,453/1,923	Ref.	Ref.	1,213/457	Ref.	Ref.
Tall height	658/563	1.10 (0.97–1.24)	1.19 (1.05–1.35)	396/170	1.26 (1.10–1.45)	1.44 (1.19–1.75)
** Body height at age 18 (*N* = 25,868)**						
Low height	1,221/972	1.03 (0.94–1.13)	1.09 (0.98–1.20)	646/213	0.99 (0.89–1.11)	0.85 (0.72–1.01)
Normal height	3,429/2,573	Ref.	Ref.	1,629/617	Ref.	Ref.
Tall height	889/745	1.03 (0.93–1.14)	1.15 (1.03–1.28)	540/240	1.05 (0.93–1.18)	1.24 (1.06–1.46)

^a^Analyzed as the interaction between height and sex and further adjusted for the main effects of parity, gestational age, parental education at birth, and equivalized household income. No remarkable changes to the estimates when adjusting for potential confounding for neither boys nor girls, and therefore, the adjusted models were solely depicted

^b^Reference categories: For explanatory variables; normal body height for age; and for outcome variables; not having reported moderate or severe spinal pain in DNBC-11 (No pain)

^c^Reference categories: For explanatory variables; normal body height for age; and for outcome variables; not having reported moderate or severe spinal pain in DNBC-18 (No pain)

Additionally, we observed that having reported spinal pain already in pre-adolescence seemed to approximately double (and for some even more) the likelihood of severe spinal pain in late adolescence independently of height at age 18 for both sexes (Supplementary File 10). For boys, the likelihood of severe spinal pain in late adolescence relative to no pain increased stepwise with increasing height at age 18 regardless of spinal pain status in pre-adolescence, which was not the case for girls. The findings were supported by Wald-tests.

### Association between distinct height trajectories and spinal pain in adolescence

**Fig. 2:**
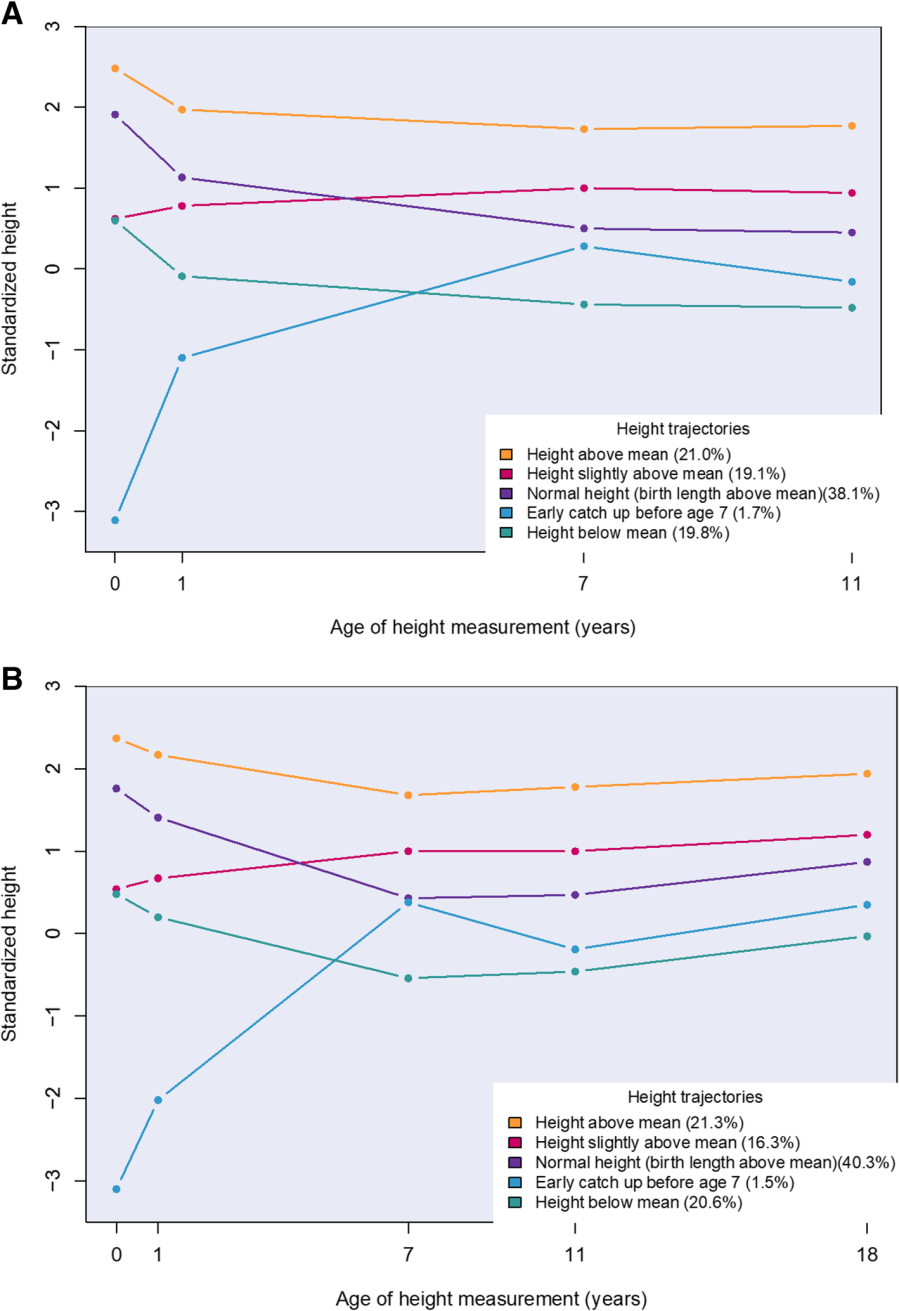
Latent growth curve modelling was used to derive distinct body height trajectories from 0 to 11 years (**A**) and from 0 to 18 years (**B**) of individuals in The Danish National Birth Cohort. The figures display the class-specific estimated average height (z-scores) for age with 95% confidence intervals. The normal height groups in both figures were characterized as having a birth length above the mean, which was expected since z-scores were made according to the WHO-reference and Scandinavian newborns are generally longer at birth

We identified five distinct height trajectories from childbirth to age 11 (Fig. 2a), as well as from childbirth to age 18 (Fig. 2b). We found girls with a height trajectory characterized by height above the mean to be 27% (RRR: 1.27, 95% CI: 1.14–1.41) more likely to have severe spinal pain in pre-adolescence and 23% (RRR: 1.23, 95% CI: 1.11–1.37) in late adolescence compared with their peers in the normal height trajectory (Table 3). Mid and low back pain were the prevailing pain regions (data not shown). Analyses of height trajectories showed no significant associations with spinal pain for boys, and we observed no tendencies of certain time periods of excessive growth that impacted on spinal pain.

**Table 3:** Adjusted relative risk ratio (RRR) of spinal pain at age 11–12 and age 18, respectively, according to height trajectory groups for both 0–11 years and 0–18 years. (The Danish National Birth Cohort, born 1996–2003)

	Girls^a,b^			Boys^a,b^		
	No. of cases Moderate/Severe	Moderate pain RRR (95% CI)	Severe pain RRR (95% CI)	No. of cases Moderate/Severe	Moderate pain RRR (95% CI)	Severe pain RRR (95% CI)
**Body height trajectories up to age 11 (***N*** = 43,765)**						
Early catch up before age 7 (1.7%)	115/45	0.89 (0.69–1.14)	0.82 (0.57–1.17)	97/33	0.96 (0.74–1.25)	1.06 (0.70–1.59)
Height below mean (19.8%)	1,424/581	0.91 (0.85–0.98)	0.88 (0.79–0.98)	1,163/348	1.02 (0.94–1.19)	0.89 (0.78–1.02)
Normal height (38.3%)	3,020/1,326	Ref.	Ref.	2,425/836	Ref.	Ref.
Height slightly above mean (19.1%)	1,094/520	1.01 (0.91–1.12)	1.15 (1.02–1.29)	1,000/336	0.96 (0.88–1.05)	0.95 (0.83–1.09)
Height above mean (21%)	1,369/666	1.13 (1.04–1.22)	1.27 (1.14–1.41)	1,280/470	1.00 (0.92–1.09)	1.07 (0.95–1.21)
**Body height trajectories up to age 18 (*N* = 26,114)** ^**c**^						
Early catch up before age 7 (1.5%)	66/54	0.70 (0.50–0.98)	0.67 (0.47–1.97)	31/14	0.77 (0.50–1.18)	0.76 (0.43–1.35)
Height below mean (20.6%)	1,218/922	0.98 (0.89–1.08)	0.99 (0.89–1.10)	569/177	1.00 (0.89–1.13)	0.77 (0.64–0.92)
Normal height (40.3)	2,475/1,834	Ref.	Ref.	1,164/463	Ref.	Ref.
Height slightly above mean (16.3%)	745/587	1.00 (0.89–1.12)	1.04 (0.91–1.17)	435/163	1.08 (0.94–1.24)	0.99 (0.82–1.22)
Height above mean (21.3%)	1,079/930	1.05 (0.95–1.16)	1.23 (1.11–1.37)	651/270	1.11 (0.99–1.25)	1.16 (0.98–1.37)

^a^Analyzed as the interaction between sex and height trajectories, and adjusted for main effects of parity, gestational age, parental education at birth and equivalized household income. No remarkable changes to the estimates when adjusting for potential confounding for neither boys nor girls, and therefore, the adjusted models were solely depicted

^b^Reference categories: For explanatory variables; normal body height trajectory for age; and for outcome variables; not having reported moderate or severe spinal pain in DNBC-11 (No pain)

^c^Reference categories: For explanatory variables; normal body height trajectory for age; and for outcome variables; not having reported moderate or severe spinal pain in DNBC-18 (No pain)

## Discussion

To our knowledge, this is the first large-scale birth cohort study including several prospectively collected height measures during childhood and adolescence as well as spinal pain in both pre-and late adolescence. We demonstrated that being tall during childhood and adolescence predisposed to spinal pain in pre-adolescence and late adolescence among girls, and tall height at age 11 and 18 predisposed to severe spinal pain among boys in late adolescence. Further, the presence of spinal pain in pre-adolescence predisposed to spinal pain in late adolescence regardless of height at age 18. Height trajectories were confirmative of tall girls being more likely to have spinal pain.

The existing literature is contradictive according to the association between height and musculoskeletal pain including back pain [35]. Our findings illustrated tall body height to be associated with spinal pain in adolescence with onset in pre-adolescence for girls and later for boys. Hebert et al. found that pubertal development and increase in height were potentially risk factors for spinal pain in 10-12-year-olds with no indication of interaction with sex [36]. In line with our findings in late adolescence, Hershkovich et al. investigated boys and girls separately and found height to be positively associated with low back pain among 17-year-old (i.e., late adolescence) girls and boys [13]. During adolescence pubertal maturation (i.e., the pubertal growth spurt and changes in hormone levels) occurs earlier in girls than in boys [7, 15, 30], and affects very differently between sexes and within individuals [15, 37]. In addition, changes in hormone levels are demonstrated to impact on pain perception [37–39]. Pubertal maturation has previously been related to back pain in adolescents [36, 40], and therefore the earlier onset of spinal pain in girls could potentially correspond the time of puberty [4], since the majority of individuals in DNBC-11 were 11 years of age. In contrast to our expectations, adjusting for pubertal maturation had no impact for boys.

Another mechanism that may explain the findings is related to growth velocity of the spine, which is around the time of pre-adolescence accelerating faster than the lower extremities. This leads to increasing height and change in body composition, which have previously been suggested to impact on back pain in adolescence [7, 41]. The back muscle strengths and the connective tissue do not develop as fast as the spine grows during adolescence, and these are important for the maintenance of the strengths of the body stature [12, 42]. Growth spurt initiates around age 10–12 in girls, whereas boys grow significant more than girls between age 11 and 18 and development of spinal pain due to height may thus be later for boys than girls [12], as also indicated in our study.

Taller stature has been associated with higher socioeconomic status and better health [43–45], whereas spinal pain seems associated with lower socioeconomic status [11]. Since taller stature was still related to spinal pain in our study also subsequent to socioeconomic adjustment, it indicated that the identified association between tall stature and spinal pain is not likely to be explained by social confounding.

### Strengths and limitations

The prospective nature of this large-scale study with access to the unique database of DNBC facilitated examination of an array of height factors from childbirth through childhood and adolescence as well as spinal pain in pre- and late adolescence. The design ensured temporality between distinct height measures and spinal pain in pre- and late adolescence. Additionally, LGCM allowed modeling of distinct height trajectories with unstructured covariance matrix i.e., height measures did not need to be reported neither at the same time or with the same interval for each individual and only one height measure per individual was required, which strengthened statistical power [34].

Some limitations to this work need to be addressed. Some of the height measures in the DNBC were either maternally reported or reported by the individual itself, which is prone to misclassification in relation to timing and accuracy. We believe, however, that misclassification of height was a minor issue since it was not assumed to be related to the reporting of spinal pain [46]. In addition, information on birth length was retrieved from The Danish Medical Birth Register, and even though the register in general holds high validity, the practical measurement of the newborn may be imprecise.

We were able to adjust for several confounders, however, the complex etiology of spinal pain including social, psychological, and biological risk factors did challenge comprehensive inclusion of confounders, and it is possible that unmeasured variables may have confounded the observed associations. For example, BMI can impact timing of pubertal maturation i.e., early overweight might result in the earlier growth spurt [47] and at the same time BMI in itself can affect the development of spinal pain [13, 48]. We did not adjust for BMI due to risk of overadjustment. Adjustment for BMI was found to have limited influence in another study [15].

Finally, as with all longitudinal birth cohort studies with long follow-up, selection into the cohort and from attrition are inevitable, and DNBC is not an exception. Compared to the source population, the participants constituted a selected sample being healthier and of higher socioeconomic position [49]. Nevertheless, methodological studies have documented the impact on effect estimates to be negligible [49, 50], and further accounting for selection by using inverse probability weighting revealed no essential changes to the findings [32, 33]. Therefore, we do not consider selection bias a major issue for the findings of this study, and thus the study population to be overall representative for individuals born in Denmark.

## Conclusion

Tall body height during childhood and adolescence is associated with spinal pain in girls in both pre- and late adolescence, and for boys by late adolescence. In addition, having had spinal pain already in pre-adolescence is remarkably increasing the likelihood of spinal pain in late adolescence regardless of height. Thus, tall height is a contributing factor to the pathogenesis of spinal pain in adolescence, with timing and mechanisms potentially being related to growth velocity, but for now this remains uncertain. Despite body height being a hardly modifiable factor, the findings from this study may from a public health perspective be important for the wider understanding of the complex interplay between risk factors of spinal pain in adolescence and further to guide the identification of vulnerable subgroups that may benefit from targeted prevention.

### Supplementary Information

**Additional file 1: Supplementary File 1. **The combination of pain frequency and intensity of the overall measure of spinal pain from DNBC-11 and DNBC-18.

**Additional file 2: Supplementary File 2. **Causal diagram of the assumed relationship between body height and spinal pain in adolescence.

**Additional file 3: Supplementary File 3. **Interpretation of relative risk ratio in multinomial logistic regression models.

**Additional file 4: Supplementary File 4. **Supplementary information on the method of latent growth curve modeling.

**Additional file 5: Supplementary File 5. **Height characteristics of girls and boys at birth, age 1, 7, 11, and 18 (The Danish National Birth Cohort, 1996-2003).

**Additional file 6: Supplementary File 6. **Prevalence of spinal pain in pre-adolescence and late adolescence.

**Additional file 7: Supplementary File 7. **Characteristics of the 90,978 participants invited to participate in DNBC-11 and included in the baseline cohort according to follow-up status at age 11 and follow-up status at age 18.

**Additional file 8: Supplementary File 8. **Adjusted relative risk ratio(RRR) of spinal pain at age 11-12 according to body height at age 7 and 11, respectively, as continuous variables in 5 cm intervals (The Danish National Birth Cohort, born 1996-2003).

**Additional file 9: Supplementary File 9. **Adjusted relative risk ratio(RRR) of spinal pain at age 11-12 according to body height at age 11, additionally adjusted for puberty maturation at age 11 (The Danish National Birth Cohort, born 1996-2003,*N* = 30,683).

**Additional file 10: Supplementary File 10. **Adjusted relative risk ratio(RRR) of spinal pain at age 18 according to body height at age 18 taking spinal pain status in pre-adolescents into account (The Danish National Birth Cohort, born 1996-2003, *N* = 25,868).

## Data Availability

The datasets generated and analyzed during the current study are not publicly available due to the data being stored and processed in the research machines of Statistics Denmark and only available for licensed institutions but are available from the corresponding author on reasonable request.

## Abbreviations

DNBC: The Danish National Birth Cohort
DNBC-11: The 11-year follow-up of The Danish National Birth Cohort
DNBC-18: The 11-year follow-up of The Danish National Birth Cohort
FPS-r: The Faces Pain Scale - revised
IPW: Inverse probability weighting
ISCED: International Standard Classification of Education
LGCM: Latent Growth Curve Modeling
NRS: The Numeric Pain Rating Scale
RRR: Relative risk ratio
SD: Standard deviation
Spinal pain: Neck pain, mid back pain and/or low back pain
YSQ: The Young Spine Questionnaire
95% CI: 95% confidence intervals

## Declarations

Written informed consent was collected from all study participants (maternally) before study participants were enrolled in the first data collection of The Danish National Birth Cohort, and again from study participants when they turned 18 years.
