# Supplementary material for: Body height and spinal pain in adolescence: a cohort study from the Danish National Birth Cohort
Source: BMC Musculoskelet Disord. 2023 Dec 11;24:958. doi: 10.1186/s12891-023-07077-3 (PMC10712045; doi:10.1186/s12891-023-07077-3)
Supplement: Supplementary file 1 — Additional file 1: Supplementary File 1. The combination of pain frequency and intensity of the overall measure of spinal pain from DNBC-11 and DNBC-18. [file 12891_2023_7077_MOESM1_ESM.docx]

**Supplementary file 1**

The combination of pain frequency and intensity of the overall measure of spinal pain from DNBC-11 and DNBC-18

*
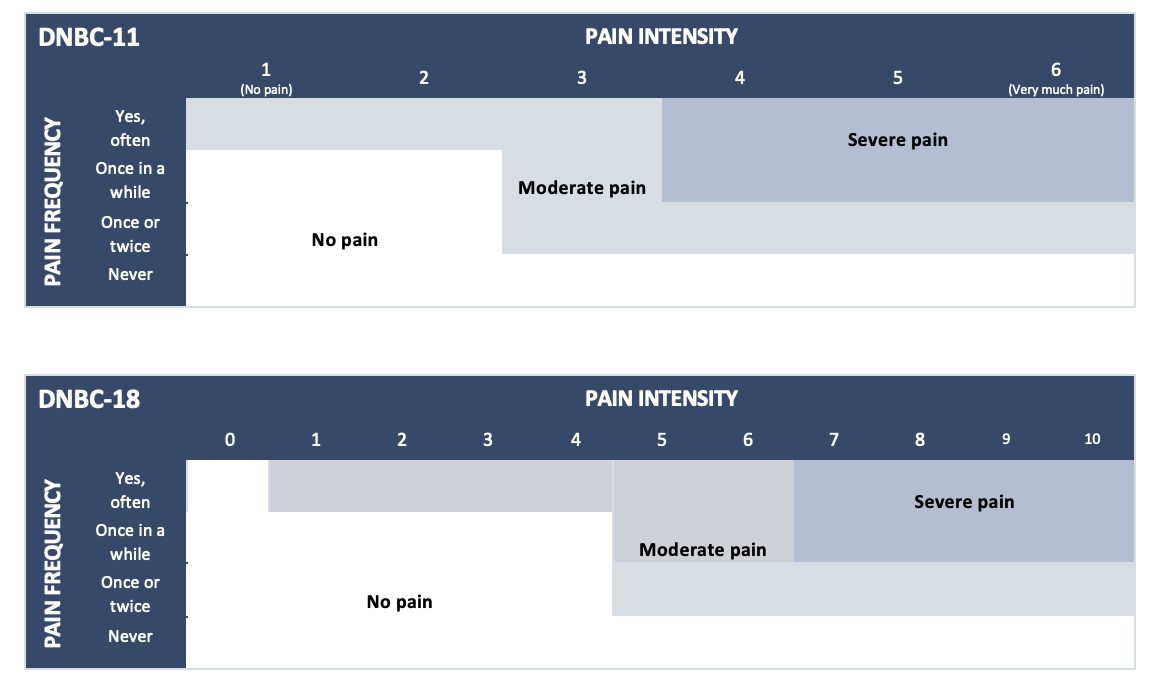
*
