# Supplementary material for: Body height and spinal pain in adolescence: a cohort study from the Danish National Birth Cohort
Source: BMC Musculoskelet Disord. 2023 Dec 11;24:958. doi: 10.1186/s12891-023-07077-3 (PMC10712045; doi:10.1186/s12891-023-07077-3)
Supplement: Supplementary file 2 — Additional file 2: Supplementary File 2. Causal diagram of the assumed relationship between body height and spinal pain in adolescence. [file 12891_2023_7077_MOESM2_ESM.docx]

**Supplementary file 2**

**Causal diagram of the assumed relationship between body height and spinal pain in**

**adolescence.**

**
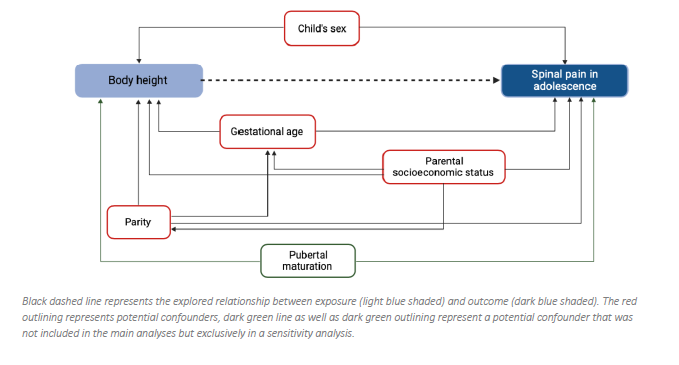
**
