# Supplementary material for: Body height and spinal pain in adolescence: a cohort study from the Danish National Birth Cohort
Source: BMC Musculoskelet Disord. 2023 Dec 11;24:958. doi: 10.1186/s12891-023-07077-3 (PMC10712045; doi:10.1186/s12891-023-07077-3)
Supplement: Supplementary file 3 — Additional file 3: Supplementary File 3. Interpretation of relative risk ratio in multinomial logistic regression models. [file 12891_2023_7077_MOESM3_ESM.docx]

**Supplementary file 3**

**Interpretation of relative risk ratio in multinomial logistic regression models**

The multinomial logistic regression model is an extension of the binomial logistic regression model and was used since the outcome variable had three categories. Children with no pain (Y=0) were considered as the reference outcome in the analyses.

For a binary covariate (Z=1 vs. Z=0) the regression coefficient b_j for a given level (Y=j) of spinal pain can then be interpreted via a relative risk ratio (RRR).

Thus, exp(b_j)=(P(Y=j|Z=1)/P(Y=j|Z=0))/(P(Y=0|Z=1)/P(Y=0|Z=0)), i.e. the risk ratio for the outcome Y=j compared to that of Y=0.
