# Supplementary material for: Body height and spinal pain in adolescence: a cohort study from the Danish National Birth Cohort
Source: BMC Musculoskelet Disord. 2023 Dec 11;24:958. doi: 10.1186/s12891-023-07077-3 (PMC10712045; doi:10.1186/s12891-023-07077-3)
Supplement: Supplementary file 4 — Additional file 4: Supplementary File 4. Supplementary information on the method of latent growth curve modeling. [file 12891_2023_7077_MOESM4_ESM.docx]

**Supplementary file 4**

**Supplementary information on the method of latent growth curve modeling**

The Latent Growth curve model assumes that the population consists of an unknown number of groups (latent classes) and that children in the same group have the same expected growth curve. The analysis was conducted as a two-step process. Firstly, using the *TRAJ package* in STATA, we estimated height trajectories by analyzing a series of models with height included as z-scores as a function of age with different pre-defined number of groups fitted as cubic curves. To evaluate the best fitting model and decide the best fitting model, different statistical indices were applied including the Akaike Information Criteria (AIC) and the Bayesian Information Criteria (BIC). In addition, we evaluated the number of individuals in each group, the shape of the trajectories and balanced these approaches against our a priori hypothesis of distinct height trajectories. A priori, we had decided to look at the different height trajectories separately for boys and girls, however, we observed a striking similarity between the two trajectory models, and decided, also supported by a likelihood ratio test, that the trajectories should not be separated by sex. Secondly, we analyzed the association between the identified trajectory groups and spinal pain in pre- and late adolescence, respectively, using multinomial logistic regression models. In sensitivity analyses, we analyzed the associations separately for each spinal region.
