# Supplementary material for: Body height and spinal pain in adolescence: a cohort study from the Danish National Birth Cohort
Source: BMC Musculoskelet Disord. 2023 Dec 11;24:958. doi: 10.1186/s12891-023-07077-3 (PMC10712045; doi:10.1186/s12891-023-07077-3)
Supplement: Supplementary file 5 — Additional file 5: Supplementary File 5. Height characteristics of girls and boys at birth, age 1, 7, 11, and 18 (The Danish National Birth Cohort, 1996-2003). [file 12891_2023_7077_MOESM5_ESM.docx]

| **Supplementary file 5**  Height characteristics of girls and boys at birth, age 1, 7, 11, and 18 (The Danish National Birth Cohort, 1996-2003) | | | |
| --- | --- | --- | --- |
|  |  | **Mean in cm (SD)** | |
| Characteristics | **N^a^**  (Girls/Boys) | **Girls** | **Boys** |
| **Birth length (N = 43,241)** |  |  |  |
| Mean (all) | 22,722/29,519 | 51.9 (2.5) | 52.7 (2.6) |
|  |  |  |  |
| Low birth length | 5,771/5,742 | 48.8 (2.2) | 49.6 (2.0) |
| Normal birth length | 14,289/12,439 | 52.4 (1.0) | 53.3 (1.1) |
| Long birth length | 2,662/2,338 | 55.6 (0.9) | 56.6 (0.9) |
|  |  |  |  |
| **Height at age 1 (N = 24,716)** |  |  |  |
| Mean (all) | 13,305/11,411 | 76.5 (2.7) | 77.9 (2.6) |
|  |  |  |  |
| Low height | 3,048/3,252 | 72.9 (1.3) | 74.7 (1.4) |
| Normal height | 8,316/6,317 | 76.8 (1.4) | 78.4 (1.1) |
| Tall height | 1,941/1,842 | 80.7 (0.9) | 81.5 (0.7) |
|  |  |  |  |
| **Height at age 7 (N = 27,189)** |  |  |  |
| Mean (all) | 13,904/13,285 | 125.6 (5.2) | 126.6 (5.3) |
|  |  |  |  |
| Low height | 2,888/2,905 | 118.5 (2.4) | 119.6 (2.6) |
| Normal height | 8,726/8,065 | 125.9 (2.5) | 126.9 (2.5) |
| Tall height | 2,290/2,315 | 133.4 (2.5) | 134.4 (2.5) |
|  |  |  |  |
| **Height at age 11 (N = 30,923)** |  |  |  |
| Mean (all) | 15,961/14,962 | 149.6 (7.2) | 149.4 (6.8) |
|  |  |  |  |
| Low height | 3,292/3,448 | 140.6 (3.1) | 139.7 (3.1) |
| Normal height | 9,976/8,906 | 149.9 (3.1) | 149.9 (3.5) |
| Tall height | 2,693/2,608 | 159.6 (3.4) | 160.6 (3.3) |
|  |  |  |  |
| **Height at age 18^b^ (N = 25,868)** |  |  |  |
| Mean (all) | 15,679/10,189 | 169.4 (6.3) | 183.5 (6.8) |
|  |  |  |  |
| Low height | 3,443/2,316 | 160.9 (2.9) | 174.6 (3.4) |
| Normal height | 9,669/5,921 | 169.9 (3.1) | 183.7 (2.9) |
| Tall height | 2,567/1,952 | 178.8 (2.7) | 193.4 (3.5) |
|  |  |  |  |
| A. Upper and lower cut-offs vary slightly from the intended 20 and 80 percentile, due to overlap in height measures (measures as integers in cm).  b. For age 18, we kept height measures both from their 18^th^ year as well as the 5,977 individuals that reported their height to be in their 17^th^ year, since growth velocity between age 17 and 18 is very limited (ref: Tinggaard J, Aksglaede L, Sørensen K, Mouritsen A, Wohlfahrt-Veje C, Hagen CP, et al. The 2014 Danish references from birth to 20 years for height, weight and body mass index. Acta Paediatrica. 2014;103(2):214-24.) | | | |
