# Supplementary material for: Body height and spinal pain in adolescence: a cohort study from the Danish National Birth Cohort
Source: BMC Musculoskelet Disord. 2023 Dec 11;24:958. doi: 10.1186/s12891-023-07077-3 (PMC10712045; doi:10.1186/s12891-023-07077-3)
Supplement: Supplementary file 6 — Additional file 6: Supplementary File 6. Prevalence of spinal pain in pre-adolescence and late adolescence. [file 12891_2023_7077_MOESM6_ESM.docx]

| **Supplementary file 6**  Prevalence of spinal pain in pre-adolescence and late adolescence | | | |  |
| --- | --- | --- | --- | --- |
|  |  | **Total** | **Girls** | **Boys** |
| **Spinal pain in pre-adolescence (N = 43,765)** | | | |  |
| No pain |  | 25,617 (58.5) | 12,839 (55.8) | 12,778 (61.5) |
| Moderate pain |  | 12,987 (29.7) | 7,022 (30.5) | 5,965 (28.7) |
| Severe pain |  | 5,161 (11.8) | 3,328 (13.6) | 2,023 (9.7) |
|  |  |  |  |  |
| **Spinal pain in late adolescence (N = 26,114)** | | |  |  |
| No pain |  | 12,267 (47.0) | 5,906 (37.3) | 6,361 (61.8) |
| Moderate pain |  | 8,433 (32.3) | 5,583 (35.3) | 2,850 (27.7) |
| Severe pain |  | 5,414 (20.8) | 4.327 (27.4) | 1,087 (10.6) |
|  |  |  |  |  |
