# Supplementary material for: Body height and spinal pain in adolescence: a cohort study from the Danish National Birth Cohort
Source: BMC Musculoskelet Disord. 2023 Dec 11;24:958. doi: 10.1186/s12891-023-07077-3 (PMC10712045; doi:10.1186/s12891-023-07077-3)
Supplement: Supplementary file 7 — Additional file 7: Supplementary File 7. Characteristics of the 90,978 participants invited to participate in DNBC-11 and included in the baseline cohort according to follow-up status at age 11 and follow-up status at age 18. [file 12891_2023_7077_MOESM7_ESM.docx]

| **Supplementary file 7**  Characteristics of the 90,978 participants invited to participate in DNBC-11 and included in the baseline cohort according to follow-up status at age 11 and follow-up status at age 18 | | | | |
| --- | --- | --- | --- | --- |
|  | **Study population with follow-up in pre-adolescence** | | **Study population with follow-up in late adolescence** | |
| Characteristics | **Study population**  43,765 (48.1) | **Lost to follow-up^a^**  47,213 (51.9) | **Study population**  26,114 (28.7) | **Lost to follow-up^a^**  64,864 (61.7) |
| Sex |  |  |  |  |
| Girls | 23,006 (52.6) | 21,422 (45.4) | 15,813 (60.6) | 28,615 (44.1) |
| Boys | 20,759 (47.4) | 25,732 (54.5) | 10,301 (39.5) | 36,190 (55.8) |
| Missing | 0 | 59 (0.1) | 0 | 0 |
| Parental educational at birthl^b^ |  |  |  |  |
| High | 27,869 (65.5) | 22,933 (48.6) | 17,277 (66.2) | 33,460 (51.6) |
| Medium | 14,869 (34.0) | 22,412 (45.4) | 8,264 (31.7) | 28,017 (43.2) |
| Low | 1,092 (2.5) | 2,793 (5.9) | 573 (2.2) | 3,312 (5.1) |
| Missing | 0 | 75 (0.2) | 0 | 75 (0.1) |
| Equivalised household income^b^ |  |  |  |  |
| 4^th^ quartile (highest) | 14,747 (33.7) | 13,247 (28.1) | 8,987 (34.4) | 19,007 (29.3) |
| 3^rd^ quartile | 12,715 (33.7) | 13,104 (27.8) | 7,527 (28.8) | 18,292 (28.2) |
| 2^nd^ quartile | 10.148 (23.2) | 11,876 (25.2) | 5,979 (22.9) | 16,045 (24.7) |
| 1^st^ quartile (lowest) | 6,155 (14.1) | 8,492 (18.0) | 3,621 (13.9) | 11,026 (17.0) |
| Missing | 0 | 494 (1.1) | 0 | 494 (0.8) |
| Parity |  |  |  |  |
| Nulliparous | 20,971 (47.9) | 21,726 (46.0) | 12,813 (49.1) | 29,884 (46.1) |
| Parous | 22,794 (52.1) | 25,427 (53.9) | 13,301 (51.0) | 34,920 (53.8) |
| Missing | 0 | 60 (0.1) | 0 | 60 (0.1) |
| Maternal age at birth |  |  |  |  |
| ≤ 25 years | 5,086 (11.6) | 7,966 (16.9) | 2,913 (11.2) | 10,137 (15.6) |
| 26-30 years | 18,669 (42.6) | 19,834 (42.0) | 11,226 (43.0) | 27,268 (42.0) |
| - 1. years | 15,032 (34.4) | 14,985 (31.6) | 9,023 (34.6) | 20,904 (32.2) |
| >35 years | 4,989 (11.4) | 4,518 (9.6) | 2,952 (11.3) | 6,555 (10.1) |
| Maternal smoking |  |  |  |  |
| Non-smoker | 36,588 (83.6) | 35,717 (75.7) | 22,012 (84.3) | 50,293 (77.5) |
| Smoking in 1. trimester | 674 (1.5) | 826 (1.8) | 397 (1.5) | 1,103 (1.7) |
| Smoker | 5,043 (11.5) | 8,879 (18.8) | 2,825 (10.8) | 11,097 (17.1) |
| Missing | 1,460 (3.3) | 1,791 (3.8) | 880 (3.4) | 2,371 (3.7) |
| Gestational age |  |  |  |  |
| Term | 41,813 (95.5) | 44,608 (94.5) | 24,978 (95.7) | 61,443 (94.7) |
| Preterm (< 37 weeks) | 1,952 (4.5) | 2,605 (5.5) | 1,136 (4.4) | 3,421 (5.3) |
| Urbanization^c^ |  |  |  |  |
| Copenhagen | 6,177 (14.1) | 5,643 (12.0) | 3,804 (14.6) | 8,016 (12.4) |
| Sub-urban Copenhagen | 4,703 (10.8) | 5,015 (10.7) | 2,701 (10.3) | 7,053 (10.9) |
| Provincial cities | 6,443 (14.7) | 6,088 (12.9) | 4,005 (15.3) | 8,526 (13.1) |
| Provincial towns | 12,667 (28.9) | 14,135 (29.9) | 7,583 (29.0) | 19,219 (29.6) |
| Rural areas | 13,759 (31.4) | 15,185 (34.3) | 8,011 (30.7) | 21,933 (33.8) |
| Missing | 16 (0.0) | 111 (0.2) | 10 (0.0) | 117 (0.2) |
| a We used chi-squared tests of heterogeneity to compare study participants with individuals lost to follow-up. Chi-squared tests were statistically significant for all variables  b Measured at the year of childbirth  c Urbanization registered at childbirth | | | | |
