# Supplementary material for: Body height and spinal pain in adolescence: a cohort study from the Danish National Birth Cohort
Source: BMC Musculoskelet Disord. 2023 Dec 11;24:958. doi: 10.1186/s12891-023-07077-3 (PMC10712045; doi:10.1186/s12891-023-07077-3)
Supplement: Supplementary file 8 — Additional file 8: Supplementary File 8. Adjusted relative risk ratio(RRR) of spinal pain at age 11-12 according to body height at age 7 and 11, respectively, as continuous variables in 5 cm intervals (The Danish National Birth Cohort, born 1996-2003). [file 12891_2023_7077_MOESM8_ESM.docx]

| **Supplementary file 8**  Adjusted relative risk ratio (RRR) of spinal pain at age 11-12 according to body height at age 7 and 11, respectively, as **continuous variables** in 5 cm intervals (The Danish National Birth Cohort, born 1996-2003) | | | | | | | |
| --- | --- | --- | --- | --- | --- | --- | --- |
|  |  | **Girls^ab^ (N = 15,576)** | |  | **Boys^ab^ (N = 14,554)** | | |
|  | **No. of cases**  Moderate/Severe | **Moderate pain**  RRR (95% CI) | **Severe pain**  RRR (95% CI) |  | **No. of cases**  Moderate/Severe | **Moderate pain**  RRR (95% CI) | **Severe pain**  RRR (95% CI) |
| **Body height at age 7**  **N = 29,138** |  |  |  |  |  |  |  |
|  |  |  |  |  |  |  |  |
| Height at age 7^c^ | 4,163/1,872 | 1.05 (1.02-1.08) | 1.15 (1.11-1.19) |  | 3,819/1,240 | 1.01 (0.99-1.04) | 1.02 (0.99-1.06) |
|  |  |  |  |  |  |  |  |
| **Body height at age 11**  **N = 29,413** |  |  |  |  |  |  |  |
|  |  |  |  |  |  |  |  |
| Height at age 11^d^ | 4,781/1,999 | 1.04 (1.02-1.06) | 1.12 (1.09-1.15) |  | 4,196/1,409 | 1.01 (0.99-1.03) | 1.05 (1.02-1.09) |
|  |  |  |  |  |  |  |  |
| a Analyzed as the interaction between height and sex, and adjusted for parity, gestational age, parental education at birth, and equivalized household income.  b Reference categories: For explanatory variables; normal body height; and for outcome variables; not having reported moderate or severe spinal pain in DNBC-11 (No pain)  c The continuous variable for height at age 7 ranges from >115 cm to => 140 cm  d The continuous variable for height at age 11 ranges from <130 cm to => 165 cm | | | | | | | |
