# Supplementary material for: Body height and spinal pain in adolescence: a cohort study from the Danish National Birth Cohort
Source: BMC Musculoskelet Disord. 2023 Dec 11;24:958. doi: 10.1186/s12891-023-07077-3 (PMC10712045; doi:10.1186/s12891-023-07077-3)
Supplement: Supplementary file 9 — Additional file 9: Supplementary File 9. Adjusted relative risk ratio(RRR) of spinal pain at age 11-12 according to body height at age 11, additionally adjusted for puberty maturation at age 11 (The Danish National Birth Cohort, born 1996-2003,N = 30,683). [file 12891_2023_7077_MOESM9_ESM.docx]

| **Supplementary file 9**  Adjusted relative risk ratio (RRR) of spinal pain at age 11-12 according to body height at age 11, additionally adjusted for **puberty maturation at age 11** (The Danish National Birth Cohort, born 1996-2003, N = 30,683) | | | | | | | |
| --- | --- | --- | --- | --- | --- | --- | --- |
|  |  | **Girls^ab^** | |  | **Boys^ab^** | | |
|  | **No. of cases**  Moderate/Severe | **Moderate pain**  RRR (95% CI) | **Severe pain**  RRR (95% CI) |  | **No. of cases**  Moderate/Severe | **Moderate pain**  RRR (95% CI) | **Severe pain**  RRR (95% CI) |
| **Body height at age 11** |  |  |  |  |  |  |  |
|  |  |  |  |  |  |  |  |
| Low height | 918/344 | 0.91 (0.84-1.00) | 0.85 (0.75-0.97) |  | 984/297 | 1.06 (0.97-1.16) | 0.94 (0.81-1.09) |
| Normal height | 2,958/1,244 | Ref. | Ref. |  | 2,437/828 | Ref. | Ref. |
| Tall height | 872/394 | 1.13 (1.02-1.24) | 1.13 (0.99-1.29) |  | 727/267 | 1.04 (0.94-1.15) | 1.12 (0.96-1.29) |
|  |  |  |  |  |  |  |  |
| a Analyzed as the interaction between height and sex and further adjusted for parity, gestational age, parental education at birth, equivalized household income and puberty stage at age 11.  b Reference categories: For explanatory variables; normal body height; and for outcome variables; not having reported moderate or severe spinal pain in DNBC-11 (No pain) | | | | | | | |
