# Supplementary material for: Body height and spinal pain in adolescence: a cohort study from the Danish National Birth Cohort
Source: BMC Musculoskelet Disord. 2023 Dec 11;24:958. doi: 10.1186/s12891-023-07077-3 (PMC10712045; doi:10.1186/s12891-023-07077-3)
Supplement: Supplementary file 10 — Additional file 10: Supplementary File 10. Adjusted relative risk ratio(RRR) of spinal pain at age 18 according to body height at age 18 taking spinal pain status in pre-adolescents into account (The Danish National Birth Cohort, born 1996-2003, N = 25,868). [file 12891_2023_7077_MOESM10_ESM.docx]

| **Supplementary file 10**  Adjusted relative risk ratio (RRR) of **spinal pain at age 18** according to body height at age 18 taking spinal pain status in pre-adolescents into account (The Danish National Birth Cohort, born 1996-2003, N = 25,868) | | | | | | | |
| --- | --- | --- | --- | --- | --- | --- | --- |
|  | **Girls^ab^** | | |  | **Boys^ab^** | | |
|  | **No. of cases**  Moderate/Severe | **Moderate pain**  RRR (95% CI) | **Severe pain**  RRR (95% CI) |  | **No. of cases**  Moderate/Severe | **Moderate pain**  RRR (95% CI) | **Severe pain**  RRR (95% CI) |
| **Height at age 18 and**  **spinal pain status at age 11^c^** |  |  |  |  |  |  |  |
|  |  |  |  |  |  |  |  |
| Low height, no spinal pain | 680/445 | 1.10 (0.97-1.23) | 1.11 (0.97-1.27) |  | 347/99 | 0.99 (0.86-1.15) | 0.78 (0.62-0.99) |
| Normal height, no spinal pain | 1,801/1,115 | Ref. | Ref. |  | 899/322 | Ref. | Ref. |
| Tall, no spinal pain | 450/346 | 1.02 (0.89-1.17) | 1.24 (1.07-1.44) |  | 309/120 | 1.14 (0.98-1.33) | 1.25 (1.00-1.57) |
| Low height, spinal pain | 541/527 | 1.77 (1.54-2.04) | 2.65 (2.28-3.07) |  | 299/114 | 1.74 (1.48-2.05) | 1.83 (1.45-2.31) |
| Normal height, spinal pain | 1,628/1,418 | 1.82 (1.66-2.01) | 2.47 (2.22-2.74) |  | 730/295 | 1.80 (1.60-2.03) | 2.03 (1.71-2.42) |
| Tall, spinal pain | 439/399 | 1.82 (1.56-2.13) | 2.57 (2.18-3.02) |  | 231/120 | 1.64 (1.37-1.96) | 2.39 (1.88-3.02) |
|  |  |  |  |  |  |  |  |
| a Analyzed as a three-way interaction between height at age 18, child’s sex and status of spinal pain at age 11 and further adjusted for parity, gestational age, parental education at birth and equivalized household income. No remarkable changes to the estimates when adjusting for potential confounding for neither boys nor girls.  b Reference categories: For explanatory variables; normal body height for age; and for outcome variables; not having reported moderate or severe spinal pain in DNBC-18 (No pain)  c Binary variable of spinal pain at age 11 (no pain vs. moderate/severe pain) | | | | | | | |
